# Evolution and Outcome of a Pediatric Pulmonary Rehabilitation Program in Hong Kong Over the Past Decade

**DOI:** 10.7759/cureus.94361

**Published:** 2025-10-11

**Authors:** Long Kiu Kelvin Chan, Shuk Kuen Chau, Wai Chong Choi

**Affiliations:** 1 Pediatrics, The Duchess of Kent Children’s Hospital at Sandy Bay, Hong Kong, HKG; 2 Pediatrics, Queen Mary Hospital, Hong Kong, HKG; 3 Physiotherapy, The Duchess of Kent Children’s Hospital at Sandy Bay, Hong Kong, HKG

**Keywords:** acute respiratory diseases, chronic respiratory diseases, multidisciplinary, pediatric, pulmonary rehabilitation

## Abstract

Background: Pulmonary rehabilitation (PR) is a well-established treatment for adults with chronic obstructive respiratory disease and other chronic respiratory diseases. Although the treatment has proved effective for pediatric asthma and cystic fibrosis, its utility for other conditions is in need of study. Since 2015, the Duchess of Kent Children’s Hospital (DKCH) in Hong Kong has been running an inpatient PR program for children with compromised lung function resulting from various underlying causes. During the lockdown amid the COVID-19 pandemic, community-based PR was developed to address patients’ needs.

Methods: We retrospectively reviewed participants in the multidisciplinary PR program from 2015 to 2024. The program included regular physiotherapy sessions that consisted of breathing training, aerobic exercises, postural correction exercises, muscle-strengthening exercises, airway clearance techniques, nutritional counseling, and disease education. Inpatient and community-based PR were employed. The lung function parameters of forced expiratory volume (FEV1) and forced vital capacity (FVC), as well as exercise capacity (as measured by the six-minute walk test (6MWT)), were assessed before and after the program.

Results: Forty-three participants were included in the study, while 11 potential participants were excluded because they were unable to perform the lung function tests. The mean age of the participants was 11.6 ± 3.8 years. The mean duration of the PR program was 68.2 ± 43.2 days. Twenty-two of the participants were referred because of chronic respiratory diseases, and 10 were referred because of deconditioning after major acute respiratory events. Their lung function parameters significantly improved (FEV1: 62.2% to 68.4%, p = 0.004, FVC: 72.6% to 79.2%, p = 0.004), as did their exercise capacity (6MWT: 472.6 m to 515.2 m; p = 0.002). Similar improvements were observed in the participants with chronic respiratory diseases (n = 22) and those with deconditioning following acute respiratory events (n = 10). The inpatient PR program resulted in significant improvements in both lung function parameters and exercise capacity, while the community-based PR improved exercise capacity. No adverse events were reported.

Conclusions: The pediatric PR program is a safe and effective treatment for children with impaired lung function or exercise capacity resulting from acute or chronic respiratory diseases. With advances in technology, community-based PR programs have become a potential alternative to inpatient programs, though further large-scale studies of the former are needed to establish their effectiveness for a broad population.

## Introduction

Pulmonary rehabilitation (PR) refers to “a comprehensive intervention based on a thorough participant assessment followed by participant-tailored therapies that include, but are not limited to, exercise training, education, and behavior change, designed to improve the physical and psychological condition of people with chronic respiratory disease and to promote the long-term adherence to health-enhancing behaviors” [1]. PR was initially developed for adults with chronic obstructive respiratory disease and has been extended to other chronic respiratory diseases, such as interstitial lung disease, bronchiectasis, and asthma [2-4].

In the pediatric population, structured PR is less commonly practiced and evaluated because of the unique disease spectrum in children and the challenges involved in designing age-appropriate exercises and programs. Studies of PR conducted for children with asthma and cystic fibrosis have had favorable outcomes [5-7]. The aim of this study was to explore whether these benefits can be extended to other chronic respiratory diseases in childhood, such as primary ciliary dyskinesia and bronchiectasis, as well as to cases of deconditioning following major acute respiratory events. The hypothesis is that children with pulmonary disease who participate in a structured PR program will demonstrate improvements in pulmonary function and exercise capacity.

The Duchess of Kent Children’s Hospital (DKCH) at Sandy Bay is a pediatric rehabilitation center in Hong Kong. A comprehensive multidisciplinary PR program has been in place there since 2015. Pediatricians, nurses, physiotherapists, and dieticians are involved in rehabilitation. The PR program includes breathing training, aerobics, postural correction, and muscle-strengthening exercises to improve endurance and airway clearance to promote mucociliary clearance, and education so that patients understand their disease, with an emphasis on compliance and nutritional counseling by dieticians. The aim of the program is to improve children’s lung function, exercise tolerance, and overall well-being. For the inpatient PR program, the participants in this study took part in physiotherapy sessions, which lasted for 30 to 60 minutes each, twice daily on two to three days per week.

With the onset of the COVID-19 pandemic in 2020-2023, and especially during lockdown, our PR program had to be modified because it became difficult for children to visit hospitals for rehabilitation. Accordingly, at the DKCH, community-based PR was developed to replace the inpatient sessions. After the first session of assessment and training by physiotherapists, the participants were instructed to perform regular home aerobic exercises, postural correction exercises, strengthening exercises, and breathing exercises using a hybrid approach combining home exercise with one or two outpatient follow-up visits each month for skill revision and reassessment of outcomes. Parents were encouraged to use exercise log sheets to reflect their adherence, which would be reviewed at each visit. This approach was in line with community-based PR programs developed in other parts of the world during the COVID-19 pandemic that proved to be feasible [8].

In this study, we retrospectively reviewed the outcomes, including lung function and exercise capacity, for the participants enrolled in the PR program. Additionally, we compared the outcomes of PR in groups with chronic respiratory diseases and those who experienced deconditioning following acute respiratory events. A comparison between inpatient and community-based PR programs was conducted, and the safety of the PR program was also assessed.

An earlier version of this article was presented as a meeting abstract at the Third Congress of the International Society of Pediatric Respiratory Diseases on June 27, 2025.

## Materials and methods

This single-center retrospective cohort study was conducted at the DKCH in Hong Kong from January 2015 to December 2024. The Institutional Review Board of the University of Hong Kong/ Hospital Authority Hong Kong West Cluster issued approval UW25-340. All of the children who participated in the PR program during the study period were recruited. The DKCH is a tertiary referral center that focuses on pediatric rehabilitation and receives referrals from across Hong Kong. Participants who could not perform the lung function or exercise capacity assessment were excluded. Those who had had less than 14 days of PR or did not undergo testing at the end of the program to reassess their lung function were also excluded. 

Doctors, physiotherapists, and other allied health professionals assessed the participants before the PR program. These assessments included clinical examinations and objective tests. Physiotherapists would prescribe appropriate exercises according to the participants' baseline functional level, with the intensity aiming to achieve a target of 70% of their maximal heart rate, calculated based on age. Adverse events were monitored with participants' reports, records of injuries, and measurements of heart rate, oxygen saturation, and respiratory rate throughout the exercise session. The lung function tests measured the forced expiratory volume in the first second (FEV1), forced vital capacity (FVC), and the distance covered in the six-minute walk test (6MWT). The same set of assessments was repeated at the end of the PR program under the same equipment, technician, and calibration procedure. The primary outcome of this study was documentation of the participants’ changes in lung function, specifically FEV1 and FVC, following the PR program. The secondary outcome was changes in the participants’ 6MWT distance. Baseline demographic data, including age, sex, underlying disease, body weight, body mass index (BMI), and z-scores relative to local growth references, were collected. The participants were categorized based on disease type as suffering from either chronic respiratory disease or deconditioning following acute respiratory events. The PR programs were differentiated as either inpatient or community-based, and the subgroup analyses compared these groups. Adverse events, such as cardiovascular events, musculoskeletal problems, or discontinuation of the PR program, were documented. All of the data were retrieved from the Clinical Data Analysis and Reporting System, an electronic database system used in our center.

The data were analyzed using IBM SPSS Statistics for Windows, Version 31 (Released 2025; IBM Corp., Armonk, New York, United States). The descriptive statistics served to assess the characteristics of the participants and the measured variables. The statistics were reported as means and standard deviations or percentages depending on the nature of the variable. Paired t-tests were conducted to examine the differences in lung function parameters and exercise results after the PR program. A subgroup analysis was performed to assess the outcome of the program for both categories of respiratory conditions and both modes of PR. All of the tests were two-tailed, and statistical significance was set at p < 0.05.

## Results

Forty-three participants were identified, of whom 11 were excluded because they were either unable to perform the lung function tests or did not perform the reassessment tests. The remaining 32 participants were included for further analysis, of whom 17 were male (53%). The mean age of the participants was 11.6 ± 3.8 years. They took part in the program for a mean duration of 68.2 ± 43.2 days. Their mean BMI was 15.4 ± 2.5 kg/m², with a z-score of -1.01. The mean body weight z-score was -0.63 when compared with the local growth reference. Eighteen of the participants underwent inpatient PR, and the other 14 took part in the community-based PR program. 

Among the participants, 22 suffered from chronic respiratory diseases, including cystic fibrosis, primary ciliary dyskinesia, non-cystic fibrosis bronchiectasis, and restrictive lung disease. The remaining 10 participants were referred when they suffered from significant deconditioning after acute respiratory events, specifically, severe acute respiratory distress syndrome (ARDS)/pneumonia (n = 9) or deconditioning after major surgery (n = 1) (Table 1).

**Table 1:** Characteristics of the study population. The data are presented as n (%) or mean ± standard deviation. ARDS: acute respiratory distress syndrome; BMI: body mass index; BW: body weight; PR: pulmonary rehabilitation.

Characteristic		Value
Mean age		11.6 ± 3.8 years
Male		17 (53.1)
Underlying diagnoses		
	Chronic respiratory disease	22 (68.8)
	(cystic fibrosis = 5,	
	primary ciliary dyskinesia = 5,	
	other non- cystic fibrosis bronchiectasis = 11,	
	restrictive lung disease = 1)	
	Deconditioning after an acute respiratory event	10 (31.3)
	(severe ARDS/pneumonia = 9,	
	and deconditioning after major surgery = 1)	
Mean BMI		15.4 ± 2.5 kg/m^2^
Mean BMI z-score		-1.01 ± 1.06
Mean BW z-score		-0.63 ± 0.92
Participation in the inpatient PR program		18 (56.3)
Mean duration of the PR program		68.2 ± 43.2 days
Mean duration of the inpatient PR program		39.8 ± 14.7 days
Mean duration of the community-based PR program		104.6 ± 40.4 days

After participation in the multidisciplinary PR program, the FEV1 and FVC of all of the participants showed significant improvement: the FEV1 increased from 62.2% to 68.4% (p = 0.004), and the FVC increased from 72.6% to 79.2% (p = 0.004). Better performance was also observed in exercise capacity as measured by the 6WMT, with the distance increasing from 472.6 m to 515.2 m (p = 0.002) (Table 2). None of the 32 participants reported an adverse event during the PR program. 

**Table 2:** Outcomes of pulmonary rehabilitation and subgroup analysis. PR: pulmonary rehabilitation; FEV1: forced expiratory volume in the first second; FVC: forced vital capacity; 6MWT: six-minute walk test.

Measurement	Pre-PR	% (age predicted)	Post-PR	% (age predicted)	Mean difference (95% confidence interval)	p-value
	Mean value		Mean value			
All participants (n = 32)						
FEV1 (n = 32)	1,247.5	62.2	1,354.1	68.4	+6.2% (2.2%-10.2%)	0.004
FVC (n = 32)	1,582.2	72.6	1,713.4	79.2	+6.7% (2.3%-11.0%)	0.004
6MWT (n = 24)	472.6 m		515.2 m		+48.9 m (19.2 m-78.6 m)	0.002
Chronic respiratory disease (n = 22)						
FEV1 (n = 22)	1,264.5	62.9	1,372.5	68.4	+5.5% (0.3%-10.7%)	0.04
FVC (n = 22)	1,614.5	74.7	1,751.5	80.6	+5.8% (0.04%-11.6%)	0.049
6MWT (n = 16)	490.1 m		533.6 m		+43.5 m (16.6 m-70.5 m)	0.004
Deconditioning after acute respiratory event (n =10)						
FEV1 (n = 10)	1,220.0	60.7	1,332.7	68.5	+7.8% (0.6%-15.0%)	0.037
FVC (n = 10)	1,552.7	67.8	1,687.3	76.3	+8.5% (1.6%-15.4%)	0.021
6MWT (n = 8)	439.9 m		520.5 m		+80.6 m (18.3 m-142.9 m)	0.018

In the subgroup analysis of those suffering from chronic respiratory disease and deconditioning following acute respiratory events, both groups showed significant improvement in terms of lung function parameters and exercise capacity. Thus, in the chronic respiratory disease group, the FEV1 increased from 62.9% to 68.4% (p = 0.04), the FVC increased from 74.7% to 80.6% (p = 0.049), and the 6MWT distance increased from 490.1 m to 533.6 m (p = 0.004). The deconditioning from the acute respiratory disease group included the nine participants with severe ARDS/pneumonia and the one patient who had had major surgery. 

After the patients participated in our PR program, their parameters improved significantly, and to an even greater extent compared with those who had chronic respiratory disease, with mean increases in the FEV1, the FVC, and the 6MWT distance of 7.8% compared with 5.5%, 8.5% compared with 5.8%, and 80.6 m compared with 43.5 m, respectively. The FEV1 increased from 60.7% to 68.5% (p = 0.037), the FVC increased from 67.8% to 76.3% (p = 0.021), and the 6MWT distance increased from 439.9 m to 520.5 m (p = 0.018) (Table 2). 

Comparing the inpatient and community-based PR programs, the mean durations were 39.8 ± 14.7 days and 104.6 ± 40.4 days, respectively. The inpatient group showed significantly improved lung function and exercise capacity, with the FEV1 increasing from 57.0% to 67.0% and a mean increase of 2.9% (p = 0.003), the FVC increasing from 68.3% to 79.2% and a mean difference of 2.7% (p ≤ 0.001), and the 6MWT distance increasing from 417.0 m to 535.1 m and a mean increase of 17.9 m (p = 0.003). In the community-based group, exercise capacity significantly improved, with the 6MWT distance increasing from 477.2 m to 517.7 m and a mean increase of 14.1 m (p = 0.021), though the lung function parameters did not differ significantly. Notably, the members of the community-based group had better lung function parameters in terms of the absolute values and percentages predicted before the PR program (Table 3).

**Table 3:** Comparison of inpatient PR and community-based PR. PR: pulmonary rehabilitation; FEV: forced expiratory volume in the first second; FVC: forced vital capacity; 6MWT: six-minute walk test.

Measurement	Pre-PR	% (age predicted)	Post-PR	% (age predicted)	Mean difference (95% confidence interval)	P-value
	Mean value		Mean value			
Inpatient group (n = 18)						
FEV1	1,649.3	57.0	1,725.7	67.0	+2.9% (3.8%-16.2%)	0.003
FVC	1,998.6	68.3	2,081.4	79.2	+2.7% (3.8%-16.5%)	< 0.001
6MWT	417.0 m		535.1 m		+17.9 m (26.8-103.4 m)	0.003
Community-based group (n = 14)						
FEV1	935.0	68.9	1,065.0	70.3	+1.9% (-2.8%-5.5%)	0.49
FVC	1,258.3	78.1	1,427.2	79.3	+2.8% (-5.9%-7.4%)	0.68
6MWT	477.2 m		517.7 m		+14.1 m (8.0 m-72.9 m)	0.021

We further performed multivariate linear regression analysis of predictors of the differences in the FEV1, FVC, and 6MWT results after the PR program. Inpatient status was a statistically significant predictor of improvement in the FEV1 and the FVC (p = 0.003 and p = 0.006, respectively), but age, sex, and disease category (chronic respiratory disease compared with deconditioning after an acute respiratory event) were not significant predictors of such improvements (Table 4).

**Table 4:** Multivariate linear regression analysis of factors that predict differences in measurements. FEV1: forced expiratory volume in the first second; vs: compared with; FVC: forced vital capacity; 6MWT: six-minute walk test.

	Factors	P-value
FEV1 change		
	Sex	0.72
	Age	0.38
	Chronic respiratory disease vs. deconditioning after an acute respiratory event	0.43
	Inpatient vs. community-based	0.003
FVC change		
	Sex	0.90
	Age	0.64
	Chronic respiratory disease vs. deconditioning after an acute respiratory event	0.43
	Inpatient vs. community-based	0.006
6MWT change		
	Sex	0.51
	Age	0.83
	Chronic respiratory disease vs. deconditioning after an acute respiratory event	0.88
	Inpatient vs. community-based	0.66

## Discussion

PR is a well-established non-pharmacological intervention for adult patients with chronic respiratory diseases, including chronic obstructive pulmonary disease and bronchiectasis [9]. In the pediatric population, researchers have explored the role of PR in treating asthma, cerebral palsy, and cystic fibrosis. For asthma patients, PR has been shown to improve lung function and quality of life [6]. For cerebral palsy patients, respiratory therapy has been shown to improve the FEV1, the FVC, and peak expiratory flow [10]. For cystic fibrosis patients, PR enhances muscle strength and respiratory muscle function, though it does not consistently improve lung function [11].

Our PR program, which is tailored to meet the needs of individual patients, incorporates approaches such as patient education, dietary management, breathing exercises, aerobic workouts, and mucociliary clearance to enhance well-being. Among the members of our cohort, irrespective of their underlying diagnoses, lung function parameters and exercise capacity improved significantly after an average of around two months of multidisciplinary PR. We observed improvements in lung function tests and exercise capacity that corresponded with meaningful clinical benefits. From our practice, most participants also reported reduced symptoms, including less shortness of breath and coughing, as well as enhanced activity levels, though it was based on subjective parents' reports. In addition, for those with chronic respiratory disease, the natural course was gradual decline in lung function; therefore, if they could result in an improvement in lung function or exercise capacity, even though small in magnitude, it would be clinically significant. Most of the participants suffered from chronic respiratory disease, and these findings are consistent with the outcomes reported in the literature on adult patients. We included participants with diagnoses that were less studied in the pediatrics literature, specifically, bronchiectasis (11 of the 32 participants) and primary ciliary dyskinesia (five of the 32 participants). The favorable outcomes suggest that PR can also be effective in these less-studied chronic respiratory conditions, particularly bronchiectasis, from which a significant portion of our cohort suffered [12,13].

On the other hand, the growth prospects for this group of participants with respiratory diseases were potentially at risk because of such factors as reduced appetite, increased work of breathing, and energy expenditure. Our cohort exhibited lower-than-average body weight and BMIs, with z-scores of -0.63 and -1.01, respectively, compared with the local pediatric population of similar age. It has been demonstrated that persistent low BMI can result in reduced lung function [14], so nutritional compromises through dietary interventions are necessary in order to optimize lung function.

Home-based exercise and tele-rehabilitation have developed rapidly in step with technological advances, and this development accelerated during the COVID-19 pandemic, when many countries were under lockdown. In a Korean study of the effectiveness of community-based PR programs, regular home-based exercise resulted in significant improvements in the participants’ lung function after three months in terms of the FEV1, peak expiratory flow, the 6MWT distance, and quality of life, with a compliance rate of 71.1% and no adverse effects reported [8]. In our cohort, only exercise capacity improved significantly in the community-based PR group, possibly because the participants assigned to community-based PR tended to have better baseline lung function than those assigned to inpatient PR, so their improvement was less remarkable. In the multivariate linear regression analysis, the inpatient mode of PR remained an independent predictor of better pulmonary function outcomes (in terms of the FEV1 and the FVC) compared with community-based PR. This result may be attributable to the fact that all of the aerobic and airway-clearance exercises were supervised by physiotherapists, though we lacked the data to assess this aspect of community-based PR. Disease severity, baseline functioning, or socioeconomic factors could be possible confounders, yet we lack the data to adjust for these. For the time being, then, inpatient PR remains the preferred treatment modality when the social conditions allow. 

The members of our cohort reported no adverse events during the PR program. Studies of chronic obstructive pulmonary disease in adults have reported extremely rare adverse events associated with PR, such as arrhythmia occurring in one of 797 patients [15]. We expect adverse events to be even less common in the pediatric population since cardiovascular disease is relatively rare in children. These previous findings and our findings alike indicate that multidisciplinary PR is a safe treatment modality for children. 

Among the limitations of our study, first, it took the form of a retrospective cohort study, with participants who had heterogeneous disease conditions and significant variation in their premorbid conditions and rehabilitation potential. For that reason, participants enrolled had different abilities in exercises, which resulted in difficulty in standardizing exercise intensity monitoring. In addition, although we were interested in improvement in their quality of life after PR, we could not evaluate this parameter because of the retrospective nature of the study, and compliance with the community-based program was not studied. On the other hand, while dietary counseling was provided for the inpatient PR participants, not all of them completed the post-PR dietary evaluation for comparison. Furthermore, the absence of a control group limited our ability to determine whether the observed improvements were attributable to PR or simply to natural recovery, particularly in the group of those experiencing deconditioning after major acute respiratory events. The apparent benefit of PR in this specific patient group merits further evaluation, for the currently available literature is limited. Lastly, this review was performed in a tertiary referral center, so the findings may not be generalizable to patients with less severe conditions. 

## Conclusions

The PR program is a safe and effective management approach that improves lung function parameters and exercise capacity. It should be considered for patients with chronic respiratory diseases and can be extended to those experiencing deconditioning following acute respiratory events. Technological advances have made community-based PR programs a potential alternative to inpatient programs, but larger-scale prospective studies are needed to establish the effectiveness of these programs across a broad population.
